# Expression of the Immune Checkpoint Protein VISTA Is Differentially Regulated by the TGF-β1 – Smad3 Signaling Pathway in Rapidly Proliferating Human Cells and T Lymphocytes

**DOI:** 10.3389/fmed.2022.790995

**Published:** 2022-02-10

**Authors:** Stephanie Schlichtner, Inna M. Yasinska, Sabrina Ruggiero, Steffen M. Berger, Nijas Aliu, Mateja Prunk, Janko Kos, N. Helge Meyer, Bernhard F. Gibbs, Elizaveta Fasler-Kan, Vadim V. Sumbayev

**Affiliations:** ^1^Medway School of Pharmacy, Universities of Kent and Greenwich, Chatham Maritime, United Kingdom; ^2^Department of Pediatric Surgery, Children's Hospital, Inselspital Bern, University of Bern, Bern, Switzerland; ^3^Department of Human Genetics, Inselspital Bern, University of Bern, Bern, Switzerland; ^4^Department of Biotechnology, JoŽef Stefan Institute, Ljubljana, Slovenia; ^5^Faculty of Pharmacy, University of Ljubljana, Ljubljana, Slovenia; ^6^Division of Experimental Allergology and Immunodermatology, Department of Human Medicine, University of Oldenburg, Oldenburg, Germany; ^7^Division of General and Visceral Surgery, Department of Human Medicine, University of Oldenburg, Oldenburg, Germany; ^8^Department of Biomedicine, University of Basel and University Hospital Basel, Basel, Switzerland

**Keywords:** immune checkpoint, VISTA, anti-cancer immunity, T lymphocytes, galectin-9

## Abstract

Immune checkpoint proteins play crucial roles in human embryonic development but are also used by cancer cells to escape immune surveillance. These proteins and biochemical pathways associated with them form a complex machinery capable of blocking the ability of cytotoxic immune lymphoid cells to attack cancer cells and, ultimately, to fully suppress anti-tumor immunity. One of the more recently discovered immune checkpoint proteins is V-domain Ig-containing suppressor of T cell activation (VISTA), which plays a crucial role in anti-cancer immune evasion pathways. The biochemical mechanisms underlying regulation of VISTA expression remain unknown. Here, we report for the first time that VISTA expression is controlled by the transforming growth factor beta type 1 (TGF-β)-Smad3 signaling pathway. However, in T lymphocytes, we found that VISTA expression was differentially regulated by TGF-β depending on their immune profile. Taken together, our results demonstrate the differential biochemical control of VISTA expression in human T cells and various types of rapidly proliferating cells, including cancer cells, fetal cells and keratinocytes.

## Introduction

Immune checkpoint proteins play crucial roles in determining the ability of human cancer cells to escape immune surveillance (1, 2). These proteins integrate into a complex machinery which is capable of blocking cytotoxic immune attacks on cancer cells by specialized human lymphoid cells and, in the long run, to fully suppress anti-tumor immunity (1, 2). These pathways can play fundamental roles and were found to be implemented by human fetal cells in order to protect the embryo against rejection by the mother's immune system (3).

With some immune checkpoint proteins, such as programmed cell death protein 1 (PD-1) and its ligand (PD-L1), cytotoxic T-lymphocyte-associated protein 4 (CTLA4), T cell immunoglobulin and mucin domain containing protein 3 (Tim-3) and its ligand galectin-9, the biochemical mechanisms underlying their expression and regulation of their biological activities have been elucidated (1, 2, 4). Others were only discovered recently and thus such mechanisms remain poorly understood. However, this is a very important issue since identification of the optimal targets for immunotherapy of cancer crucially hinges on our understanding of the biochemistry of immune checkpoint pathways responsible for immune escape. One of such immune checkpoint proteins is V-domain Ig-containing suppressor of T cell activation (VISTA) which plays a crucial role in the suppression of human T cell responses during cancer progression (5–7). VISTA is expressed mainly in blood cells and plays a complex role in regulating immune responses (5–7). Myeloid cells show the highest levels of expression, but lymphoid cells, especially T lymphocytes also express this protein where it can be used as a receptor to suppress their anti-cancer activities (5–7). Interestingly, VISTA has been reported to display both receptor and ligand properties (5–7). However, its receptors (when it acts as a ligand) remain to be identified. One of the VISTA ligands is VSIG3 (V-set and immunoglobulin domain containing 3), which is a member of the immunoglobulin superfamily and is highly expressed in human brain and testis (8). Another VISTA ligand is galectin-9, which is a member of galectin family of proteins conserved throughout evolution (9). Galectin-9 contains two similar, but not identical, subunits bound through a peptide linker, which can be of different size (9, 10). There are three isoforms of galectin-9 which vary in linker size (10). Galectin-9 isoforms can interact with VISTA on the surface of cytotoxic T cells and induce programmed death most likely through prevention of granzyme B release from these cells. Granzyme B is a proteolytic enzyme used to induce apoptotic death of target cells, and its activation inside cytotoxic T cells, which fail to release it, is followed by their programmed death (9). Several types of human cancer cells are known to express VISTA too (11, 12). However, the mechanisms which regulate VISTA expression in human cells remain unknown. By analyzing the promoter region of human VISTA, we noticed that it contains response elements for Smad3 transcription factor which is activated by human transforming growth factor beta type 1 (TGF-β) through specific plasma membrane-associated receptors. Interestingly, we have recently reported that the TGF-β-Smad3 pathway is involved in regulating galectin-9 expression in human cancer and embryonic cells (3). TGF-β displays both autocrine and paracrine activities and is able to induce its own expression, which is also Smad3-dependent as is the expression of galectin-9 (3). Thus, in both cancer and embryonic cells, this pathway is self-controlling and self-sustaining. In this work we aimed to study whether the TGF-β-Smad3 pathway is also involved in VISTA expression.

We discovered for the first time that VISTA expression is controlled by the TGF-β-Smad3 signaling pathway. Interestingly, in T lymphocytes, VISTA is only upregulated by TGF-β if they do not display cytotoxic activity (lack granzyme B expression), while if T cells express this proteolytic enzyme and display cytotoxic activity, VISTA expression is decreased in the presence of TGF-β. We hypothesized that this phenomenon could be possibly triggered by differential nuclear compartmentalisation of VISTA encoding gene (VSIG).

## Materials and Methods

### Materials

RPMI-1640 medium for cell culture, fetal bovine serum, supplements and basic laboratory chemicals were purchased from Sigma (Suffolk, UK). Microtiter plates for ELISA were obtained from Oxley Hughes Ltd (London, UK). Rabbit antibodies against VISTA, galectin-9, granzyme B, phospho-S423/S425-Smad3, Smad4 and TRIM33 as well as goat anti-rabbit horseradish peroxidase (HRP) labeled secondary antibody were purchased from Abcam (Cambridge, UK). Antibodies against β-actin were purchased from Proteintech (Manchester, UK). Goat anti-mouse and anti-rabbit fluorescently-labeled dye secondary antibodies were obtained from Li-COR (Lincoln, NE, USA). Mouse anti-Smad3 antibody, ELISA-based assay kits for the detection of VISTA, galectin-9 (both kits contain mouse capture antibodies against VISTA and galectin-9, respectively) and TGF-β as well as human recombinant TGF-β1 protein and mouse anti-Smad3 antibody were purchased from Bio-Techne (R&D Systems, Abingdon, UK). Anti-Tim-3 mouse monoclonal antibody was described before (13). All other chemicals employed in this study were of the highest grade of purity commercially available.

### Cell Lines and Primary Human Samples

Cell lines used in this work were purchased from either the European Collection of Cell Cultures, American Tissue Culture Collection or CLS Cell Lines Service GmbH. Cell lines were accompanied by identification test certificates. Wilms Tumor cell line WT3ab was kindly provided by Dr. C. Stock (Children's Cancer Research Institute, Vienna, Austria) and cultured as it was previously described (14).

Jurkat T, MCF-7, THP-1, WT-3ab, HaCaT keratinocytes and K562 were cultured in RPMI 1640 media supplemented with 10% fetal bovine serum, penicillin (50 IU/ml), and streptomycin sulfate (50 μg/ml).

TALL-104 CD8-positive cytotoxic T lymphocytes, derived from human acute lymphoblastic leukemia (TALL), were cultured according to the ATCC instructions. Briefly, ATCC-formulated Iscove's Modified Dulbecco's Medium was used. To make the complete growth medium we added 100 units/ml recombinant human IL-2; 2.5 μg/ml human albumin; 0.5 μg/ml D-mannitol and fetal bovine serum to a final concentration of 20% (15). Medium was also supplemented with penicillin (50 IU/ml), and streptomycin sulfate (50 μg/ml).

Primary human AML mononuclear blasts (AML-PB001F, newly diagnosed/untreated) were also purchased from AllCells (Alameda, CA, USA) and handled according to the manufacturer's recommendations. The studies were performed following ethical approval (REC reference: 16-SS-033).

Placental tissues (CVS, chorionic villus sampling) and amniotic fluids were collected after obtaining informed written consent from pregnant women at the University Hospital Bern, Inselspital. Fetal cells were handled and cultured as described before (3, 16).

Primary human T cells where isolated from buffy coat blood (purchased form the Deutsches Rotes Kreuz and following ethical approval from the Medizinische Ethikkommission der Carl von Ossietzky Universität Oldenburg) using Ficoll-density centrifugation. PBMCs were collected and T cells purified using a commercial CD3 T cell negative isolation kit (Biolegend) according to the manufacturer's protocol. 200,000 T cells per 200 μl were incubated for 16 h with and without TGF-β at a final concentration of 2 ng/ml in RPMI medium.

### Western Blot Analysis

VISTA, granzyme B, galectin-9, Tim-3, phospho-S423/S425 Smad-3, Smad4 and TRIM33 were measured by Western blot and compared to the amounts of β-actin (protein loading control), as previously described (17).

Li-Cor goat secondary antibodies conjugated with infrared fluorescent dyes, were used as described in the manufacturer's protocol for visualization of specific proteins (Li-Cor Odyssey imaging system was employed). Western blot data were quantitatively analyzed using Odyssey software called Image Studio and values were subsequently normalized against those of β-actin.

### Chromatin Immunoprecipitation (ChIP)

ChIP was performed as described recently (18). Resting Jurkat T cells and those treated for 24 h with 2 ng/ml TGF-β were subjected to the study. 5 × 10^6^ cells were used for immunoprecipitation. Cross-linking was performed using 1.42% formaldehyde followed by quenching with 125 mM glycine for 5 min. Cells were then washed twice with PBS and subjected to ChIP in accordance with ChIP-IT high sensitivity kit (Active Motif) protocol. Immunoprecipitation was performed using mouse monoclonal anti-Smad3 antibody (R&D Systems, Abingdon, UK), and IgG isotype control antibody was used for a negative control IP. The Smad3 epitope recognized by this antibody does not overlap with DNA and co-activator binding sites of this protein. Immunoprecipitated DNA was then purified and subjected to quantitative real-time PCR (qRT-PCR) which was performed as outlined below. The following primers were designed using NCBI Primer-Blast primer designing tool: forward – 5′-GCCTACCACATACCAAGCCC-3′ and reverse: 5′-ATCGGCAGTTTAAAGCCCGT-3′. These primers allow to amplify the fragment of the promoter region of VSIG (VISTA gene), which surrounds Smad3-binding sites.

### qRT-PCR Analysis

To detect VISTA mRNA levels, we used qRT-PCR (15). Total RNA was isolated using a GenElute™ mammalian total RNA preparation kit (Sigma-Aldrich) according to the manufacturer's protocol, followed by reverse transcriptase–polymerase chain reaction (RT-PCR) of a target protein mRNA (also performed according to the manufacturer's protocol). This was followed by qRT-PCR. The following primers were used. VISTA: forward – 5′-GATGCACCATCCAACTGTGT-3′, reverse – 5′-GCAGAGGATTCCTACGATGC-3′; actin: forward – 5′-TGACGGGGTCACCCACACTGTGCCCATCTA-3′, reverse – 5′-CTAGAAGCATTTGCGGTCGACGATGGAGGG-3′. Reactions were performed using a LightCycler® 480 qRT-PCR machine and SYBR Green I Master kit (Roche, Burgess Hill, UK). The assay was performed according to the manufacturer's protocol. Values representing VISTA mRNA levels were normalized against those of β-actin.

### On-Cell Western Analysis

Cell surface levels of VISTA protein were analyzed using on-cell Western analysis performed using a Li-COR Odyssey imager and the assay was performed in line with manufacturer's recommendations as previously described (9).

### Enzyme-Linked Immunosorbent Assays (ELISAs)

Secreted VISTA and TGF-β were measured in cell culture medium (VISTA was also measured in some of the cell lysates), by ELISA using R&D Systems kits (see Section Materials) according to manufacturer's protocols. To study recruitment of co-activators Smad4 and TRIM33 by Smad3 we used ELISA-based assay where we applied mouse anti-Smad3 antibody (R&D Systems) as capture. The plate was coated with this antibody (1:500) overnight followed by blocking with 1% BSA (dissolved in phosphate buffered saline, PBS). Then cell lysates were applied and incubated for 2 h followed by 5 times washing with TBS (50 mM Tris–HCl, 140 mM NaCl, pH 7.3) containing 0.1% Tween 20 (TBST). Rabbit anti-TRIM33 or anti-Smad4 antibodies were used (1:1000, 2h incubation) to detect these proteins interacted with Smad3. Finally, the plates were washed 5 times with TBST and horseradish peroxidase (HRP) labeled goat anti-rabbit antibody was applied for 1 h at room temperature. The plates were washed 5 times with TBST followed by visualization through the peroxidase reaction (ortho-phenylenediamine/H_2_O_2_). The reactions were quenched after 10 min with an equal volume of 1 M H_2_SO_4_ and the color development was measured in a microplate reader as the absorbance at 492 nm. Schematically both ELISA formats are illustrated in Supplementary Figure 1.

### Statistical Analysis

Each experiment was performed at least three times and statistical analysis, was conducted using a two-tailed Student's *t*-test. Statistical probabilities (p) were expressed as ^*^ when *p* < 0.05; ^**^ when *p* < 0.01 and ^***^ when *p* < 0.001.

## Results

**Figure 1:**
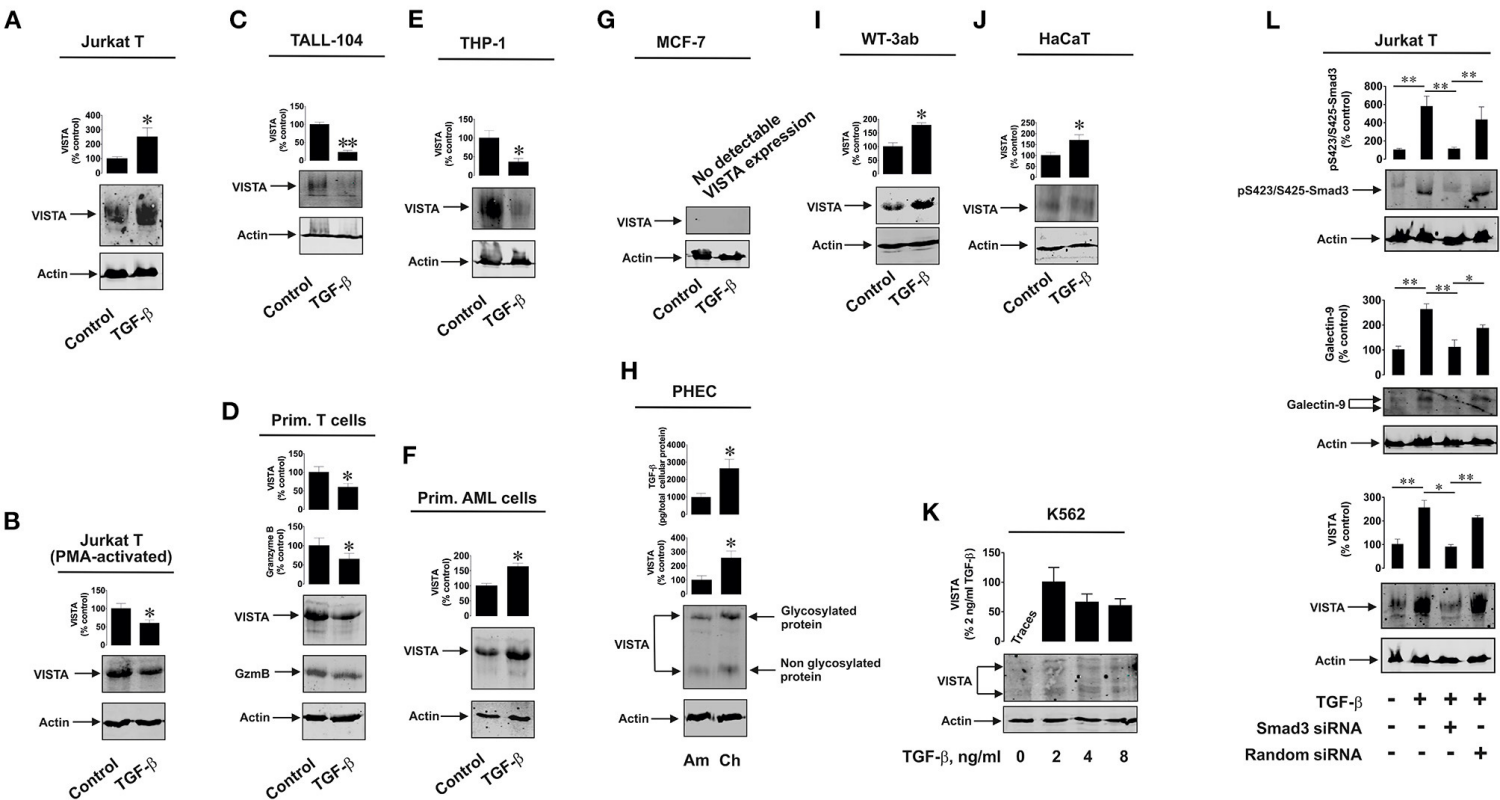
Differential effects of TGF-β on VISTA expression in human T lymphocytes and various rapidly proliferating cell types. Resting Jurkat T cells **(A)** and those activated for 24 h with 100 nM PMA **(B)**, TALL-104 cytotoxic T cells **(C)**, primary human T lymphocytes **(D)**, THP-1 human AML cells **(E)** and primary human AML blasts **(F)** as well as MCF-7 breast cancer cells, **(G)** WT-3ab Wilms tumor cells **(I)**, non-malignant HaCaT keratinocytes **(J)** were exposed for 24 h (primary T cells – for 16 h) to 2 ng/ml TGF-β followed by Western blot detection of VISTA expression as outlined in Materials and Methods. In primary human T cells **(D)**, granzyme B protein expression was also analyzed by Western blot. Expression of VISTA and TGF-β secretion were tested in primary human embryonic cells **(H)** obtained during amnion (ca week 20) and chorion (weeks 13-14) stages. K562 cells **(K)** expressing barely detectable amounts of VISTA were exposed to 2, 4 or 8 ng/ml TGF-β for 24 h followed by Western blot analysis of VISTA expression. Finally, resting and Smad3 knockdown Jurkat T cells as well as those transfected with random siRNA (negative control siRNA) were exposed to 2 ng/ml TGF-β for 24 h followed by Western blot detection of phospho-Smad3, galectin-9 and VISTA **(L)**. Images are from one experiment representative of 4-7 which gave similar results. Quantitative data represent mean values ± SEM of 4-7 independent experiments. * *p* < 0.05 and ** *p* < 0.01 vs. control.

Firstly, we observed whether TGF-β could induce VISTA expression in human T cells, since this protein was found to mediate galectin-9-induced apoptosis in cytotoxic T lymphocytes (9). We used non-treated Jurkat T cells (CD4-positive), which express just traces of granzyme B protein (9, 16), as well as PMA-activated (activation was performed for 24 h using 100 nM PMA) Jurkat T cells which express granzyme B protein (9, 16). Cells were exposed for 24 h to 2 ng/ml TGF-β followed by detection of VISTA expression by Western blot analysis. We found that TGF-β significantly upregulated VISTA expression in resting Jurkat T cells (Figure 1A) while, in PMA-activated cells, VISTA levels were reduced by exposure to TGF-β compared to the non-treated cells (Figure 1B). In both cell types TGF-β induced phosphorylation of Smad3 transcription factor (Supplementary Figure 2A). We then tested cytotoxic CD8-positive TALL-104 cells. Similarly to granzyme B-expressing PMA-activated Jurkat T cells (Figure 1B), TGF-β (2 ng/ml for 24 h) reduced VISTA expression in TALL-104 cells (Figure 1C). However, Smad3 phosphorylation was upregulated by TGF-β (Supplementary Figure 2B right panel). The expression levels of granzyme B were downregulated by TGF-β in TALL-104 cells (Supplementary Figure 2B, right panel), which is in line with a previous observation reporting TGF-β-induced downregulation of granzyme B expression in cytotoxic T cells (19). Importantly, TALL-104 cells expressed Tim-3 and rather small amounts of galectin-9 (Supplementary Figure 2B, left panel). Expression of both proteins was not affected by TGF-β (Supplementary Figure 2B, left panel). We then isolated primary human T cells (all CD3 positive cells were isolated to allow for the presence of CD8-positive cytotoxic T cells, CD4-positive helper type T cells and CD4-positive cytotoxic cells). These cells were then exposed to 2 ng/ml TGF-β for 16 h (given the higher reactivity of primary T cells compared to T cell lines). Both VISTA and granzyme B expressions were lowered by TGF-β in these cells (Figure 1D). Primary human T cells expressed both Tim-3 and galectin-9 (Supplementary Figure 2C), but galectin-9 levels were very low (Supplementary Figure 2C). Smad3 phosphorylation was highly upregulated by TGF-β in primary T cells. We then tested human myeloid leukemia cells, which are known to express high levels of VISTA. We used the THP-1 cell line (monocytic leukemia) and more premature primary human acute myeloid leukemia (AML) blasts. In THP-1 cells, TGF-β is known to highly upregulate galectin-9 expression in a Smad3-dependent manner (a significant increase in Smad3 phosphorylation induced by TGF-β was also reported for these cells) (3, 20). Furthermore, THP-1 cells do not express detectable amounts of granzyme B protein and do not show detectable catalytic activity of this enzyme (9). However, TGF-β downregulated VISTA expression in THP-1 cells (Figure 1E). On the other hand, in primary AML blasts, VISTA expression was significantly upregulated by TGF-β (Figure 1F). Given the variation in the effects observed, we investigated whether TGF-β can induce VISTA expression in the cells, which in a resting state do not express this protein. We investigated MCF-7 human epithelial breast cancer cells, where TGF-β was reported to trigger the expression of galectin-9 (3) and discovered that TGF-β was unable to induce even traces of VISTA expression (Figure 1G). VISTA mRNA was also barely detectable in these cells by qRT-PCR (Supplementary Figure 3).

Interestingly, primary human embryonic cells express VISTA protein which is also present on their surface (16). The earlier the stage of pregnancy, the more TGF-β fetal cells produce (3). We compared primary human fetal cells taken at the amnion stage (*ca*. week 20) and chorion stage (weeks 13–14). In line with our previously reported results (3, 16), cells obtained at the chorion stage released significantly higher levels of TGF-β and expressed higher VISTA levels (Figure 1H). Also, we reported earlier that Smad3 phosphorylation levels are significantly higher in fetal cells obtained at chorion stage (3). Some solid tumor cells also express VISTA, which may be upregulated by TGF-β. We found that in human Wilms tumor (a type of pediatric kidney tumor) cells WT-3ab, which express VISTA, TGF-β significantly upregulated its expression (Figure 1I). These cells expressed small amounts of Tim-3 but higher amounts of galectin-9. Galectin-9 expression in these cells was upregulated by TGF-β, as in other cancer cells studied in the past (3), and this correlated with TGF-β-induced Smad3 phosphorylation (Supplementary Figure 4).

The same effect applied to non-malignant, rapidly proliferating, human keratinocytes (HaCaT). These cells express VISTA, and this expression was upregulated by 24 h exposure to 2 ng/ml TGF-β [Figure 1J; Smad3 activation in these cells takes place as well, though differently from the one reported for cancer and embryonic cells (3)]. Interestingly, in lymphoblasts isolated from human chronic myelogenous leukaemia (K562), which express traces of VISTA, this expression (unlike in MCF-7 cells where no VISTA protein expression is detected at all) can be induced by 24 h of exposure to 2 ng/ml TGF-β where this concentration appears to be the most effective (Figure 1K). Higher TGF-β concentrations (4 and 8 ng/ml) also induced VISTA expression, but the expression level was not higher compared to those observed with exposure to 2 ng/ml TGF-β. Resting K562 cells were reported to show undetectable amounts of phospho-Smad3, but this was highly upregulated by TGF-β (3).

Interestingly, most of the investigated cell types display glycosylated VISTA with a molecular weight of *ca*. 52 kDa [this phenomenon was discussed previously (9)], while others have some partially glycosylated (K562, Figure 1K) or non-glycosylated (molecular weight *ca*. 30 kDa; primary human embryonic cells, Figure 1H) VISTA. Since biologically functional VISTA is known to be glycosylated [52 kDa (9)], these results suggest that the glycosylation velocity of this protein may vary depending on the cell type. It is also possible that primary human embryonic cells store certain amounts of non-glycosylated VISTA, though the reason for this remains to be understood.

We then asked whether TGF-β-induced VISTA expression is Smad3-dependent or not. We used resting Jurkat T cells which we transfected with Smad3 siRNA (or random siRNA – negative control). Successful knockdown (in terms of biological effect) was monitored by Western blot analysis of intracellular levels of phosphorylated Smad3. Expressions of both VISTA and galectin-9 were induced by TGF-β and attenuated by Smad3 siRNA but not random siRNA (Figure 1L). This suggests that the process is Smad3-dependent.

**Figure 2:**
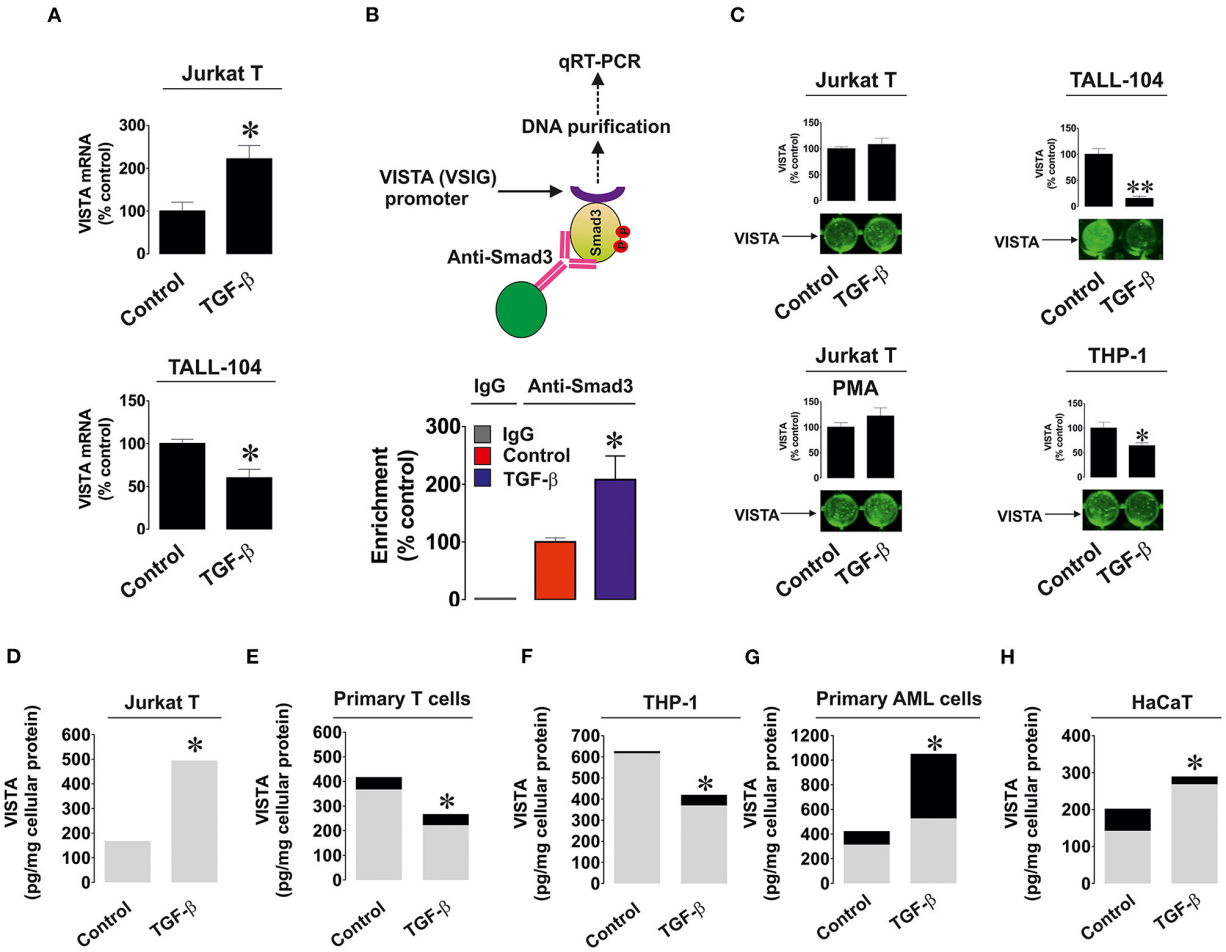
Effects of TGF-β on expression and distribution of VISTA in various human cell types. **(A)** Jurkat T and TALL-104 cells were exposed to 2 ng/ml TGF-β for 24 h followed by total RNA isolation and detection of VISTA mRNA using qRT-PCR as outlined in Materials and Methods; **(B)** Resting Jurkat T cells and those treated for 24 h with 2 ng/ml TGF-β were subjected to ChIP followed by qRT-PCR as described in Methodology section in order to detect whether Smad3 binds to the VSIG promoter region; **(C)** Resting as well as PMA-activated Jurkat T cells, TALL-104 and THP-1 cells were exposed for 24 h to 2 ng/ml TGF-β followed by detection of cell surface-based VISTA by on-cell Western (see Materials and Methods for details); Jurkat T cells **(D)**, primary human T lymphocytes **(E)**, THP-1 AML cells **(F)**, primary AML blasts **(G)** and HaCaT keratinocytes **(H)** were exposed to 2 ng/ml TGF-β for 24 h followed by detection of cell-associated (gray color) and secreted (black color) VISTA levels. Images are from one experiment representative of 5 which gave similar results. Data are the mean values of 5 independent experiments. * *p* < 0.05 vs. control (total VISTA).

We investigated whether the observed effects are taking place at mRNA level given the fact that TGF-β-regulated VISTA expression appeared to be Smad3 (transcription factor)-dependent. We used resting and TGF-β-treated (2 ng/ml for 24 h) Jurkat T (where TGF-β upregulates VISTA expression) and TALL-104 cells (where TGF-β treatment downregulated VISTA expression) and measured VISTA mRNA levels as outlined in Materials and Methods. We found that in Jurkat T cells TGF-β significantly upregulated VISTA mRNA levels while in TALL-104 we observed significant downregulation (Figure 2A). To verify that Smad3 binds VSIG (VISTA gene) directly we used ChIP qRT-PCR which confirmed that this process does take place and TGF-β significantly increased the fold of enrichment (Figure 2B) confirming that Smad3 can directly interact with the VSIG promoter region.

Importantly, we tested whether TGF-β impacted the cell surface presence and secretion of VISTA. We detected VISTA on the cell surface of resting and PMA-activated Jurkat T cells, TALL-104 and THP-1 cells with or without exposure to 2 ng/ml TGF-β for 24 h. The results suggested when the total amounts of VISTA were strongly downregulated (TALL-104 and THP-1 cells), VISTA surface presence was reduced. In other cases, TGF-β did not significantly impact VISTA cell surface presence (Figure 2C), suggesting that this growth factor mainly controls VISTA expression but not its distribution within the cell. We also measured both cell-associated and secreted protein using ELISA. We tested Jurkat T cells (Figure 2D), primary human T cells (Figure 2E), THP-1 cells (Figure 2F), primary human AML cells (Figure 2G), and HaCaT keratinocytes (Figure 2H). Other cell types reported in Figure 1 did not secrete VISTA. The mean values ± SEM of cell-associated and secreted VISTA are shown in Supplementary Table 1. We observed that TGF-β only significantly affected VISTA secretion (upregulation) in AML cells, whereas total expression levels remained in line with our Western blot observations (Figures 2D–H and see also Figures 1A–K for comparison). This also suggests that TGF-β itself is unlikely to impact VISTA secretion in any of the cell types.

**Figure 3:**
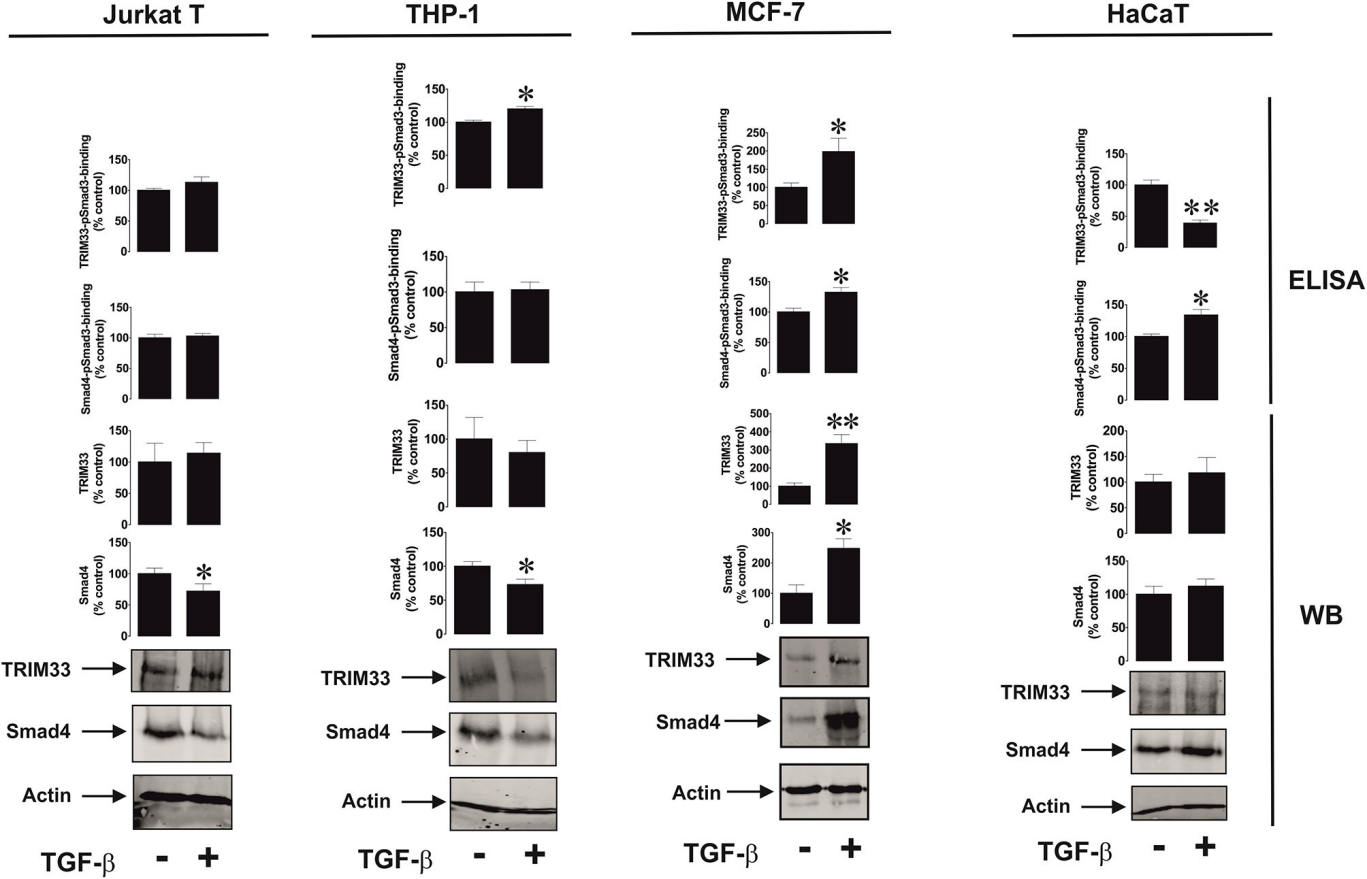
Effects of TGF-β on Smad4 and TRIM-33 levels and phsopho-Smad3-Smad4 or phsopho-Smad3-TRIM33 interactions in a variety of rapidly proliferating human cells. Jurkat T, THP-1, MCF-7 and HaCaT cells were exposed to 2 ng/ml TGF-β for 24 h followed by Western blot detection of Smad4/TRIM-33 levels as well as ELISA-based analysis of phospho-Smad3-Smad4 or phospho-Smad3-TRIM33 interactions. Images are from 1 experiment representative of 4 which gave similar results. Quantitative data represent mean values ± SEM of 4 independent experiments. * *p* < 0.05 and ** *p* < 0.01 vs. control.

We then assessed if the differential effects observed could be due to involvement of different Smad3 co-activators – TRIM33 (tripartite motif-containing protein 33), also known as transcriptional intermediary factor 1 gamma (TIF-1γ), and Smad4 (21). TRIM33 is known mainly to interact with Smad3 in order to induce expression of repressed genes (21), while Smad4 is used to trigger expression of non-repressed target genes. We tested resting Jurkat T cells (VISTA expression is upregulated by TGF-β), THP-1 (downregulation of VISTA expression by TGF-β), MCF-7 (no effect, since the cells do not express detectable amounts of VISTA) and HaCaT (where VISTA expression is upregulated by TGF-β). We also tested the recruitment of both co-activators by Smad3 using an ELISA-based assay (see Section Materials and Methods and Supplementary Figure 1 for details). We found that there was no specific correlation between the effect of TGF-β on VISTA expression and the amounts of Smad4/TRIM33 accumulated in the cells or recruited by Smad3 (Figure 3). This suggests that the effects observed are unlikely to be due to the involvement of differential co-activators in different cell types.

## Discussion

VISTA has recently been reported to actively participate in the suppression of anti-cancer cytotoxic immune responses of T cells (5, 9). However, the mechanisms underlying the expression of this crucial immune checkpoint protein remain unknown. The promoter region of the VISTA gene, VSIR, contains several Smad response elements. As such, we hypothesized that the TGF-β-Smad3 pathway could be responsible for inducing VISTA expression. The experiments showed that TGF-β-induced Smad3 activation led to an increase in VISTA expression in various cell types including resting CD4-positive Jurkat T cells, primary human AML blasts derived from myeloid cell precursors, primary human embryonic cells, Wilms tumor cells, chronic AML cells and HaCaT keratinocytes. In other cell types studied – PMA-activated, granzyme B expressing Jurkat T cells with cytotoxic activity, cytotoxic CD8-positive TALL-104 cells, primary CD3-positive human T lymphocytes (in both cell types, the overall granzyme B expression level is high) and monocytic AML THP-1 cells [which, unlike cytotoxic T cells, do not express detectable amounts of granzyme B protein (9)] – VISTA expression was reduced.

The biological reason for the observed downregulatory effects in various types of T cells described above is most likely their biological function associated with cytotoxic activity (granzyme B expression is used as a marker of their cytotoxic activity). As we have recently reported, galectin-9 interacts with VISTA on the surface of granzyme B-expressing T cells, which leads to leakage of granzyme B from intracellular granules, resulting in its activation (9). As such, these T cells may undergo programmed death mediated by VISTA. As seen in Figure 1D and Supplementary Figure 2, granzyme B expression is reduced by exposure of the cells to TGF-β, which is in line with previous observations (19). With monocytic AML cells, which do not express detectable amounts of granzyme B protein (9), this biological response has probably more complex reasons. THP-1 cells secrete high levels of galectin-9, especially when pre-treated with PMA or other triggers of exocytosis (such as latrotoxin, or Toll-like receptor (TLR) ligands) (20). They are also capable of secreting VISTA. Importantly, secretion of both proteins may be required to suppress cytotoxic immune attack conducted by T cells. However, a certain ratio of the amounts of galectin-9 and VISTA secreted is important to achieve the immunosuppressive effect, as we have recently shown (9). TGF-β induces galectin-9 expression in THP-1 cells but not its secretion (3). Upregulation of VISTA expression could potentially lead to increased level of its translocation onto the cell surface with possible shedding, leading to increased levels of soluble VISTA (the evidence of such an effect can be seen in Figure 2F). As a result, this kind of response could be required to sustain an effective ratio of secreted galectin-9 and VISTA proteins.

Importantly, TGF-β was only able to induce VISTA expression in cells which already express detectable amounts of this protein. If cells did not express VISTA (MCF-7), no TGF-β-dependent induction was observed (as it can be seen from Figure 1G). Smad3 was obviously responsible for the process of TGF-β-induced VISTA expression. Knock-down of Smad3 expression by siRNA led to attenuation of TGF-β-induced VISTA expression in Jurkat T cells (Figure 1L). Further experiments demonstrated that the observed effects take place on both protein and mRNA levels. Furthermore, Smad3 was found to directly bind to the VSIG (VISTA gene) promoter region using ChIP followed by qRT-PCR (Figures 2A,B).

However, it is necessary to understand how such a differential effect of TGF-β on VISTA expression can be achieved biochemically. The activities of the Smad3 co-activators Smad4 and TRIM33 did not appear to determine any differences in response (Figure 3). Clearly, some cells use more Smad4 leading to reduction in its quantity, without substantially affecting the production and recruitment of TRIM33 by Smad3. This applies to resting Jurkat T cells and THP-1 cells, where the effects of TGF-β on VISTA expression were opposing. MCF-7 upregulate the amounts and usage of both co-activators, while VISTA expression was not induced in these cells. HaCaT cells, which respond to exposure to TGF-β by upregulation of VISTA expression, showed increased Smad3-Smad4 interaction activity and decreased TRIM33 recruitment induced by TGF-β (Figure 3).

These results suggest that the differential activities of Smad4 and TRIM33 are unlikely to contribute to achieving responses with TGF-β-induced changes in VISTA expression in various cell types. Importantly, VISTA expression is most likely to be activated by Smad3 in partnership with Smad4. TRIM33 is involved in Smad3-dependent de-repression of target genes (21). As shown in the Figure 1G, in MCF-7 cells, where VISTA gene VSIR is most likely repressed, TGF-β failed to induce its expression. The same is most likely to apply to galectin-9, where the TGF-β-Smad3 pathway was able to upregulate even very low expression levels of this protein (3).

Importantly, the receptors recognizing TGF-β could be internalized by the cell when complexed to its ligand (22–24). Furthermore, the amount of active TGF-β receptor (TGFBR) molecules on the cell surface could be increased in the presence of the ligand. This phenomenon was reported for several types of receptors including TGFBRs (22–24) and Toll-like receptors (TLRs) (25).

**Figure 4:**
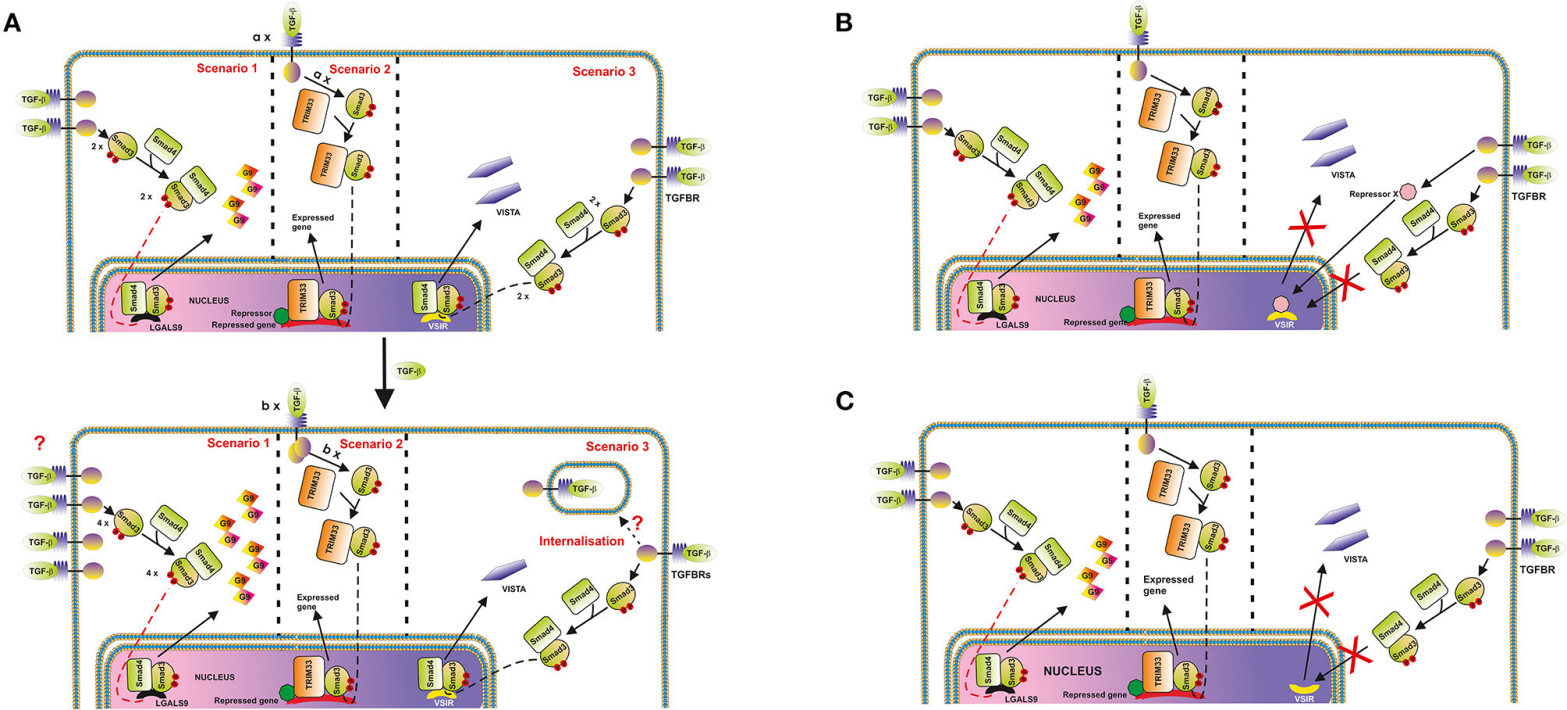
Possible mechanisms underlying the differential regulation of protein (and in particular, VISTA) expression by the TGF-β-Smad3 pathway. **(A)** Changes in cell surface-based TGFBR levels. An example where galectin-9 expression is upregulated, and VISTA is downregulated is shown. The key message of this scheme is that the expression of target proteins could be differentially regulated by the TGF-β-Smad3 pathway, recruiting Smad4/TRIM33 co-activators. Differential responses are possibly determined by alterations of TGFBR levels on the surface of target cells. In the second scenario, “a” and “b” represent numbers of respective protein molecules. **(B)** Possibility of activation of a hypothetical protein-repressor of VSIG is depicted; **(C)** TGF-β-triggered changes in the localization of VSIG-containing locus during nuclear compartmentalisation in cell types where VISTA is downregulated by TGF-β (most likely scenario).

Thus, one could hypothesize that in cells where VISTA is upregulated, the respective number of active TGFBR molecules on the cell surface at each time point may either increase upon stimulation with TGF-β or remain unchanged. Conversely, in cells where VISTA is downregulated, the number of appropriate TGFBR molecules may be reduced. As such, the number of VISTA molecules produced will decrease. This proposed regulatory pathway is depicted in Figure 4A. However, TGF-β-induced Smad3 activation which took place regardless its effect on VISTA expression, which suggests that the strategy described above is unlikely to be involved.

On the other hand, one cannot rule out the involvement of repressing transcription factors like ATF1, which is known to participate in TGF-β-induced Smad3-dependent downregulation of granzyme B expression (19). Specifically to ATF1, unlike the granzyme B gene promoter region, the promoter region of VSIR (a gene which encodes VISTA) does not have ATF1 response elements [CREB/ATF response elements ACGTAA or ACGTCC (19)]. If the cells express ATF1, and granzyme B, TGF-β will downregulate expression of this enzyme, however, with VISTA the effect is differential. This mechanism is shown in the Figure 4B.

In our view, the most likely molecular mechanism underlying the observed cell function-dependent differential impact of TGF-β on VISTA expression is associated with nuclear compartmentalisation (chromatin re-organization). This phenomenon has recently been investigated and thoroughly discussed as a fundamental molecular mechanism underlying regulation of gene expression (26). Importantly, this kind of regulation approach was reported for T cell development (26, 27). The loci containing genes, the expression of which needs to be downregulated or repressed can be re-located to the periphery of the nucleus, while active genes are normally biased toward the nuclear interior (26). As such, in the cells, which would benefit from downregulation of VISTA expression in response to the presence of TGF-β, respective loci may be re-localized accordingly, while other TGF-β-Smad3 inducible genes [like LGALS9 encoding galectin-9, which is upregulated by TGF-β in THP-1 cells (3) while VISTA is downregulated] can remain active. This kind of re-location to the periphery and possible association with nuclear lamina is sufficient to downregulate expression of respective genes (26). This regulatory strategy is presented in the Figure 4C.

The ability of T cell subsets that do not express granzyme B protein to respond to TGF-β by increasing VISTA expression may be the crucial biochemical mechanism used by granzyme B-negative T cell lymphoma/leukemia cells. Apoptotic T cells, which are always present in such cases, release TGF-β (15, 28). TGF-β could then induce VISTA expression which suppresses cytotoxic T lymphocytes trying to attack malignant T cells.

Taken together, our results have uncovered the biochemical phenomenon of differential control of VISTA expression in human T cells and various types of rapidly proliferating cells, including several types of cancer cells, fetal cells and keratinocytes. These results indicate the involvement of a complex molecular mechanism controlling expression of the critical immune checkpoint protein known as VISTA. Activation of this regulatory pathway could lead to differential outcomes which are most likely determined by the specific cell functions and type of interaction with immune and target cells.

## Data Availability Statement

The original contributions presented in the study are included in the article/Supplementary Material, further inquiries can be directed to the corresponding author/s.

## Ethics Statement

The studies involving human participants were reviewed and approved by Research Ethics Committee (REC). Primary human AML mononuclear blasts (AML-PB001F, newly diagnosed/untreated) obtained from AllCells (Alameda, CA, USA) were used following ethical approval (REC reference: 16-SS-033). Primary human T cell work received ethical approval from the Medizinische Ethikkommission der Carl von Ossietzky Universität Oldenburg. Placental tissues and amniotic fluids were collected after obtaining informed written consent from pregnant women at the University Hospital Bern, Inselspital following ethical approval. The patients/participants provided their written informed consent to participate in this study.

## Author Contributions

SS performed majority of the experiments together with IY. EF-K, SR, MP, and JK contributed to performing the experiments on cytotoxic T cells. NM and BG performed isolation and experiments on primary human T cells. NA, EF-K, and SB completed the work with primary embryonic cells. VS designed the study and planned all the experiments together with EF-K and BG and analyzed the data. VS, EF-K, and BG wrote the manuscript. All authors contributed to the article and approved the submitted version.

## Conflict of Interest

The authors declare that the research was conducted in the absence of any commercial or financial relationships that could be construed as a potential conflict of interest.

## Publisher's Note

All claims expressed in this article are solely those of the authors and do not necessarily represent those of their affiliated organizations, or those of the publisher, the editors and the reviewers. Any product that may be evaluated in this article, or claim that may be made by its manufacturer, is not guaranteed or endorsed by the publisher.
