# Supplementary material for: Expression of the Immune Checkpoint Protein VISTA Is Differentially Regulated by the TGF-β1 – Smad3 Signaling Pathway in Rapidly Proliferating Human Cells and T Lymphocytes
Source: Front Med (Lausanne). 2022 Feb 10;9:790995. doi: 10.3389/fmed.2022.790995 (PMC8866318; doi:10.3389/fmed.2022.790995)
Supplement: Supplementary file 1 [file Data_Sheet_1.docx]

**SUPPLEMENTARY INFORMATION**

**Expression of the Immune Checkpoint Protein VISTA is Differentially Regulated by the TGF-β1 – Smad3 Signalling Pathway in Rapidly Proliferating Human Cells and T Lymphocytes**

Stephanie Schlichtner, Inna M. Yasinska, Sabrina Ruggiero, Steffen M. Berger, Nijas Aliu, Mateja Prunk, Janko Kos, N. Helge Meyer, Bernhard F. Gibbs, Elizaveta Fasler-Kan, Vadim V. Sumbayev


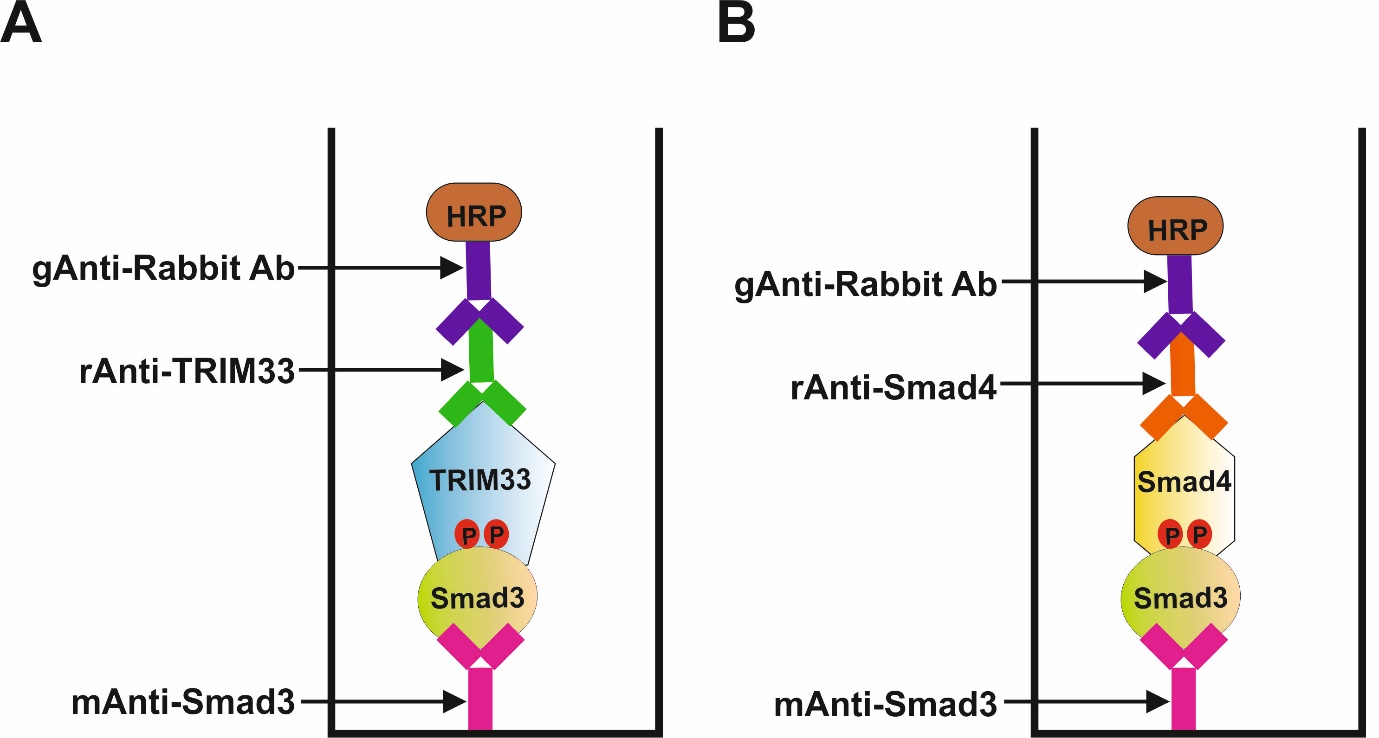


**Supplementary figure 1. Scheme of Smad3-co-activator binding assays.** The scheme outlines the ELISA-based approaches to analyse the interactions of Smad3 and TRIM33 (A) as well as Smad3 and Smad4 in cell lysates.


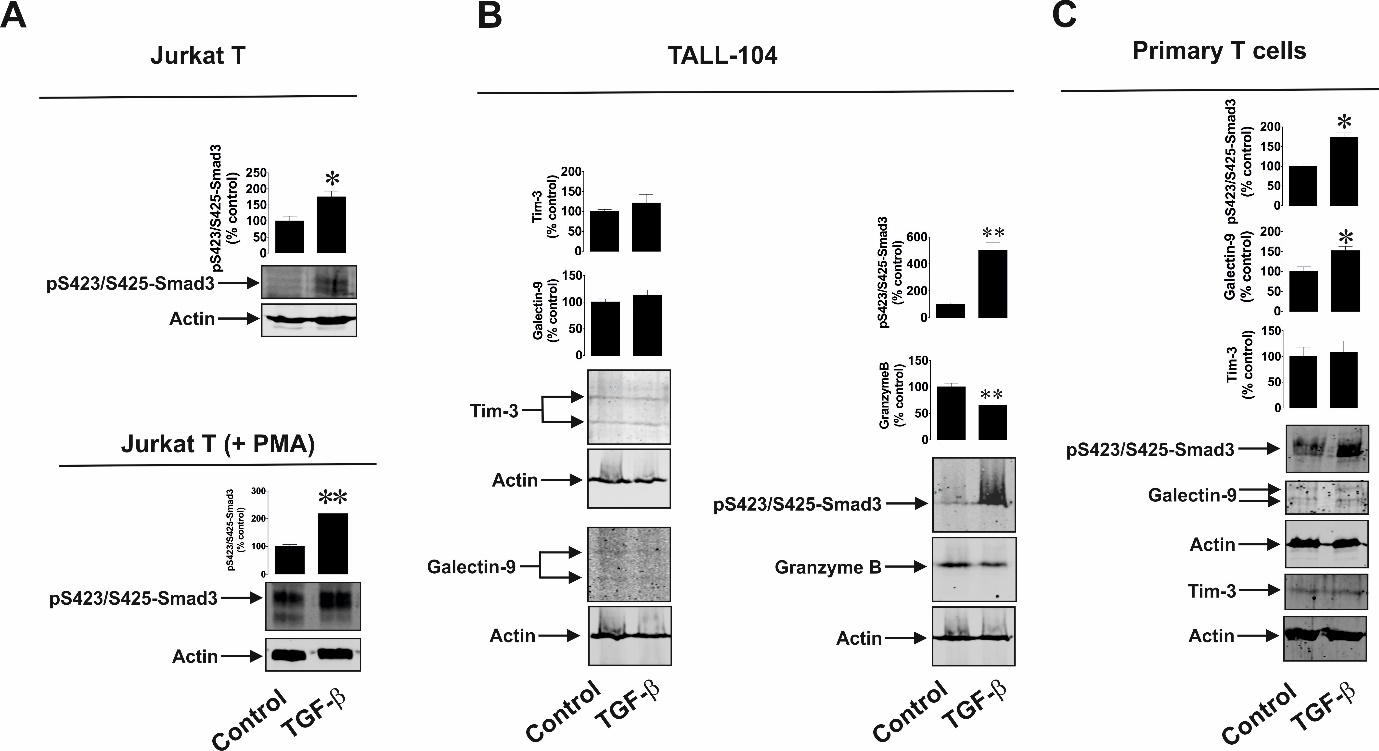


**Supplementary figure 2. Effects of TGF-β on Smad3 phosphorylation, expression of granzyme B, Tim-3 and galectin-9 in various types of human T cells.** Resting (A, top panel) and PMA-activated (A, bottom panel) Jurkat T cells, TALL-104 cells (B) and primary human T lymphocytes (C) were exposed for 24 h to 2 ng/ml TGF-β followed by Western blot detections of indicated proteins. Images are from 1 experiment representative of 4 which gave similar results. Quantitative data represent mean values ± SEM of 4 independent experiments. * - p < 0.05 and ** - p < 0.01 *vs* control.


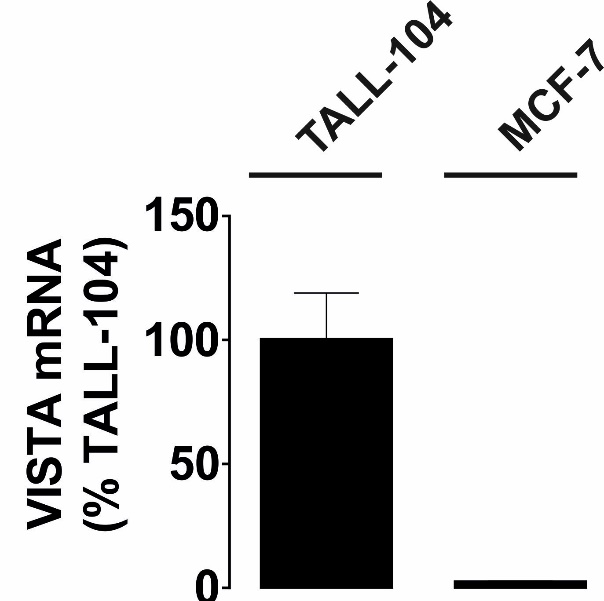


**Supplementary figure 3. VISTA mRNA levels in MCF-7 and TALL-104 cells.** VISTA mRNA levels were measured in TALL-104 (positive control) and MCF-7 cells by qRT-PCR as outlined in Materials and Methods. Data are the mean values ± SEM of 3 independent experiments.


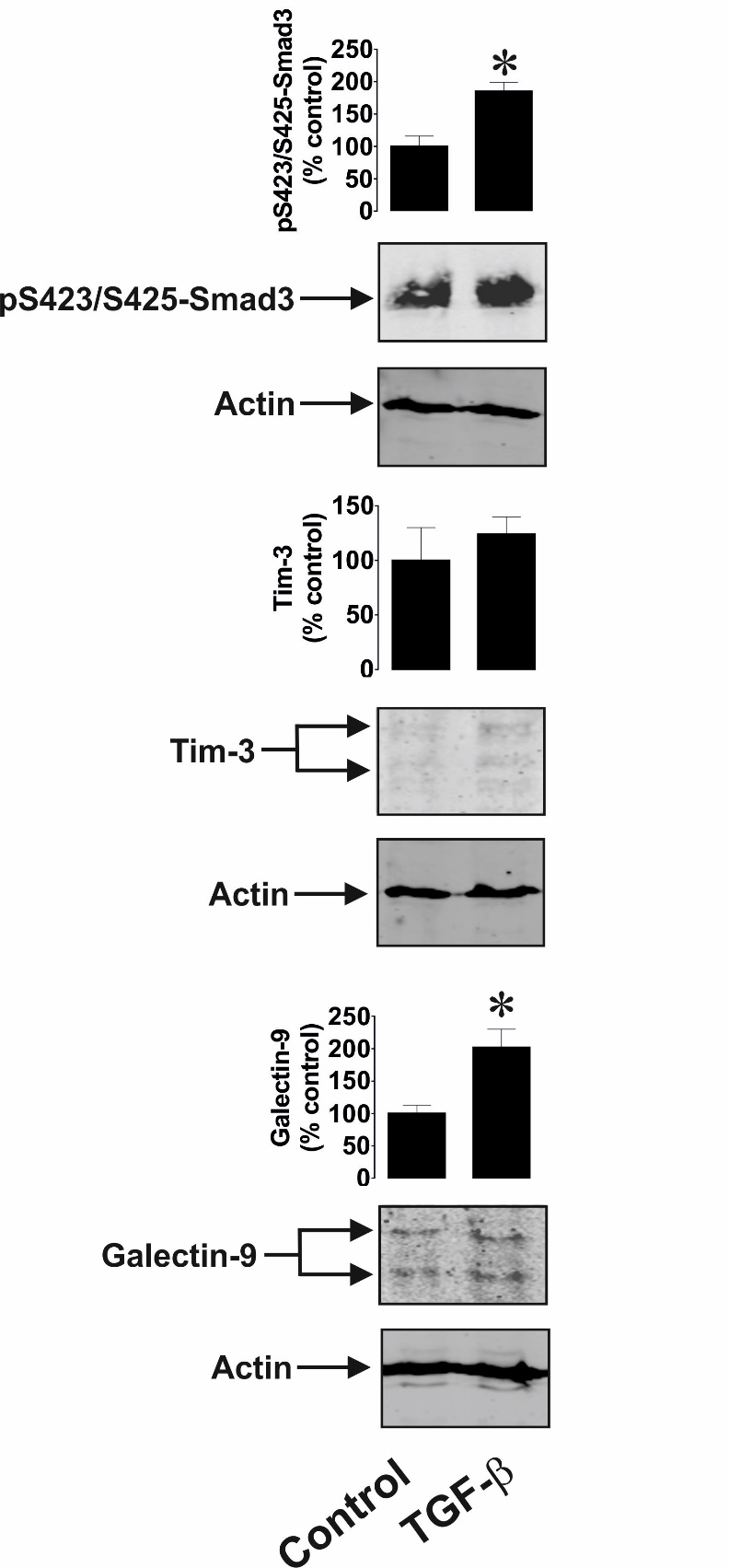


**Supplementary figure 4. Effects of TGF-β on Smad3 phosphorylation, expression of granzyme B, Tim-3 and galectin-9 in WT-3ab Wilms tumor cells.** WT-3ab cells were exposed for 24 h to 2 ng/ml TGF-β followed by Western blot detections of indicated proteins. Images are from 1 experiment representative of 4 which gave similar results. Quantitative data represent mean values ± SEM of 4 independent experiments. * - p < 0.05 *vs* control.

**Supplementary table 1. Levels of secreted and cell-associated VISTA protein in various human cell types (values presented in the Figure 2 D – H.** Ctrl – means “control”, ND – non detectable, NA – not applicable.

| **Cells**  **Protein** | **Jurkat T** | | **Primary**  **T cells** | | **THP-1** | | **Primary**  **AML cells** | | **HaCaT** | |
| --- | --- | --- | --- | --- | --- | --- | --- | --- | --- | --- |
|  | **Ctrl** | **TGF-β** | **Ctrl** | **TGF-β** | **Ctrl** | **TGF-β** | **Ctrl** | **TGF-β** | **Ctrl** | **TGF-β** |
| **VISTA**  **(cell-associated),**  **pg/mg cell protein** | **166**  **±**  **12** | **492**  **±**  **52** | **365**  **±**  **54** | **220**  **±**  **18** | **614**  **±**  **58** | **367**  **±**  **27** | **310**  **±**  **36** | **522**  **±**  **64** | **143**  **±**  **18** | **266**  **±**  **29** |
| **VISTA**  **(secreted),**  **pg/mg cell protein** | **ND**  **±**  **NA** | **ND**  **±**  **NA** | **50**  **±**  **7** | **45**  **±**  **4** | **10**  **±**  **2** | **55**  **±**  **8** | **108**  **±**  **17** | **524**  **±**  **73** | **59**  **±**  **8** | **21**  **±**  **3** |
